# Near-Infrared Emitting PbS Quantum Dots for in Vivo Fluorescence Imaging of the Thrombotic State in Septic Mouse Brain

**DOI:** 10.3390/molecules21081080

**Published:** 2016-08-18

**Authors:** Yukio Imamura, Sayumi Yamada, Setsuko Tsuboi, Yuko Nakane, Yoshikazu Tsukasaki, Akihito Komatsuzaki, Takashi Jin

**Affiliations:** 1 Laboratory for Nano-Bio Probes, Quantitative Biology Center (QBiC), Riken, Suita 565-0874, Japan; sayumi.yamada@riken.jp (S.Y.); setsuko.tsuboi@riken.jp (S.T.); yuko_nakane@digital-biology.co.jp (Y.N.); ytsuka0214@gmail.com (Y.T.); komatsu.qbic@gmail.com (A.K.); 2 Tomy Digital Biology Co. Ltd., Tokyo 110-0008, Japan; 3 Immunology Frontier Research Center (IFReC), Osaka University, Suita 565-0871, Japan

**Keywords:** near-infrared fluorescence, in vivo imaging, quantum dots, pathological state, lipopolysaccharide, sepsis

## Abstract

Near-infrared (NIR) fluorescent imaging is a powerful tool for the non-invasive visualization of the inner structure of living organisms. Recently, NIR fluorescence imaging at 1000–1400 nm (second optical window) has been shown to offer better spatial resolution compared with conventional NIR fluorescence imaging at 700–900 nm (first optical window). Here we report lead sulfide (PbS) quantum dots (QDs) and their use for in vivo NIR fluorescence imaging of cerebral venous thrombosis in septic mice. Highly fluorescent PbS QDs with a 1100 nm emission peak (QD1100) were prepared from lead acetate and hexamethyldisilathiane, and the surface of QD1100 was coated with mercaptoundecanoic acid so as to be soluble in water. NIR fluorescence imaging of the cerebral vessels of living mice was performed after intravascular injection (200–300 μL) of QD1100 (3 μM) from a caudal vein. By detecting the NIR fluorescence of QD1100, we achieved non-invasive NIR fluorescence imaging of cerebral blood vessels through the scalp and skull. We also achieved NIR fluorescence imaging of cerebral venous thrombosis in septic mice induced by the administration of lipopolysaccharide (LPS). From the NIR fluorescence imaging, we found that the number of thrombi in septic mice was significantly increased by the administration of LPS. The formation of thrombi in cerebral blood vessels in septic mice was confirmed by enzyme-linked immunosorbent assay (ELISA). We also found that the number of thrombi significantly decreased after the administration of heparin, an inhibitor of blood coagulation. These results show that NIR fluorescence imaging with QD1100 is useful for the evaluation of the pathological state of cerebral blood vessels in septic mice.

## 1. Introduction

For the past decade, a variety of quantum dots (QDs) that that emit from the visible to near-infrared (NIR) region have been developed for bioimaging probes [1]. The main advantages of QDs used for fluorescence imaging are their high brightness with emission tunability, multiplexed excitation, and their stability for long-term observation. Owing to these superior properties, QD probes are widely used for bioimaging in vitro and in vivo [1,2,3,4,5]. Compared with conventional organic dyes and fluorescent probes, QDs are much brighter probes in the NIR region over 700 nm. Recently, we have demonstrated that highly fluorescent NIR QDs that emit at the second optical window (1000–1400 nm) are useful for non-invasive deep-tissue imaging of lymph nodes, breast tumor, and cerebral blood vessels in living mice [6,7,8,9]. Among the several types of NIR fluorescent probes that have been reported, such as single-walled carbon nanotubes [10,11,12,13,14] and rare earth nanoparticles [15], lead sulfide (PbS) QDs are the brightest probes in the second optical window [9,16]. In this work, we show that PbS QDs can be used as NIR fluorescent probes for the evaluation of the pathological state of cerebral blood vessels after whole-body inflammation (i.e., sepsis) [17].

In the diseased brain, several lines of research have suggested the potential validity of nanoparticle application to neurological diagnosis [18,19] and treatment of brain diseases [20,21]. Sepsis causes systemic inflammatory response syndrome, a devastating condition in the whole body [22]. When septic encephalopathy occurs in the brain, the symptoms include coma, delirium, and cognitive dysfunction, and are considered important social issues to be solved in patients in intensive care and critical care units [23,24]. Although various imaging technologies (e.g., computed tomography, magnetic resonance imaging, etc.) can be applied to address and uncover pathophysiological state, the pathophysiology of septic encephalopathy is unknown [25]. In the brain vascular system, however, it has been reported that the improvement of the brain microvascular environment (e.g., microvascular thrombus) is important for better outcomes of septic encephalopathy [26].

The microvascular thrombus is formed in disseminated intravascular coagulation and in systemic inflammatory response syndrome [27]. Molecular mechanisms of the microvascular thrombus can be explained by the following processes: (1) increased tissue factor [28]; (2) thrombomodulin inhibition [29]; and (3) inhibition of plasminogen [30]. These processes, followed by ischemia, remarkably aggravate disseminated intravascular coagulation, which leads to multiple organ failure and increases the mortality rate of septic patients [31]. Conversely, heparin (which was used in this study) inhibits the tissue factor and alleviates disseminated intravascular coagulation [32,33]. Non-invasive visualization of thrombi in the brain is important in order to clarify the mechanism of disseminated intravascular coagulation in septic encephalopathy. In this study, we examined whether PbS QDs that emit at the second optical window could visualize cerebral vessel thrombosis in a mouse model of sepsis. Our findings show that PbS QDs can act as novel and potential probes to determine the pathological state of sepsis in the brain.

## 2. Results and Discussion

### 2.1. Characteristics of NIR Fluorescent PbS QDs

**Figure 1:**
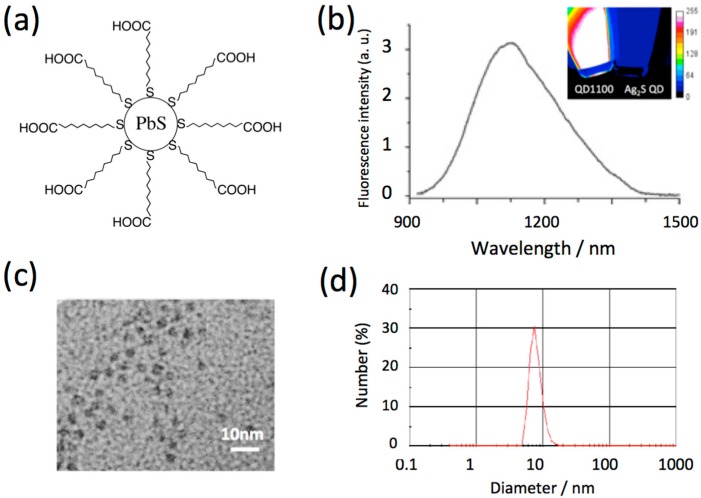
(**a**) Schematic structure of highly-fluorescent lead sulfide (PbS) quantum dots (QD) with emission peak at 1100 nm (QD1100). QD1100 is coated with mercaptoundecanoic acid to make it soluble in water; (**b**) Fluorescence spectrum of QD1100 in water. Inset: near-infrared (NIR) fluorescence image (>1000 nm) of QD1100. For comparison, fluorescence image (>1000 nm) of silver sulfide (Ag_2_S) QDs (emission maximum: 1250 nm) is also shown. Excitation: 785 nm; (**c**) TEM image of QD1100; (**d**) Hydrodynamic diameter of QD1100 in water, determined by dynamic light scattering.

Although single-walled carbon nanotubes, silver sulfide (Ag_2_S) QDs, and rare earth-doped nanoparticles emit at the second optical window, their fluorescence quantum yields (QY) are much less than that of PbS QDs [9]. In this work, we used highly-fluorescent PbS QDs [6] (QD1100, QY = 8% in water) with an emission peak of 1100 nm for in vivo imaging of cerebral blood vessels. The PbS QDs were prepared by the reaction of lead chloride and hexadimethyldisilathiane in a mixture of oleylamine and oleic acid [6]. Since the resulting PbS QDs are coated with oleylamine, their surface is hydrophobic, and so the PbS QDs are not soluble in water. To modify the QDs to be hydrophilic, their surface was modified with mercaptoundecanoic acid (Figure 1a) [6]. The mercaptoundecanoic acid-coated QDs are easily dispersed in the water phase. Figure 1b shows the NIR fluorescence spectrum of QD1100 (in water), where its spectral width is ca. 250 nm. The inset shows the fluorescence image of QD1100 excited at 785 nm. For comparison, the fluorescence image of glutathione-coated Ag_2_S QDs [9] is also shown. The fluorescence of QD1100 is at least ten times brighter than that of the Ag_2_S QDs. Transmission electron microscope (TEM) image shows the crystallinity of QD1100, with its diameter of less than 10 nm (Figure 1c). The hydrodynamic diameter of QD1100 in water was estimated to be 7.5 ± 2.5 nm by using dynamic light scattering (Figure 1d). The aqueous solution of QD1100 was very stable at pH > 8 at room temperature, and aggregation of the QDs was not observed over the course of one month.

### 2.2. Biocompatibility of NIR Fluorescent PbS QDs

**Figure 2:**
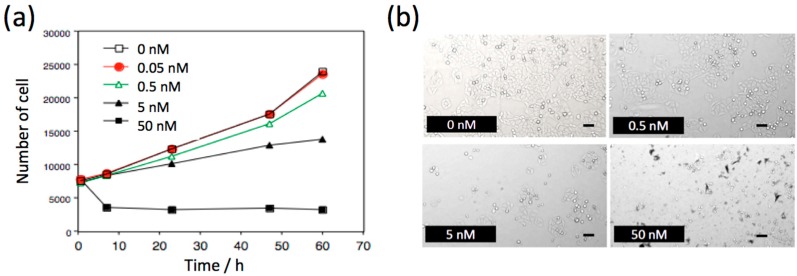
(**a**) Time course of the proliferation of HeLa cells in the absence and presence of QD1100. (**b**) Bright field images of HeLa cells cultured after 60 h in the absence and presence of 0.5, 5, and 50 nM of QD1100. Scale bar: 100 μm.

To check the biocompatibility of NIR fluorescent PbS QDs, we examined the effect of QDs on the proliferation of HeLa cells (Figure 2). With increasing concentration of PbS QDs, the rate of cell proliferation was decreased in a dose-dependent manner. However, at a QD concentration of less than 5 nM, the cell proliferation rate was slightly increased within 6 h, and the number of cells increased with culturing times. While at the high concentration (50 nM) of PbS QDs, the number of cells decreased by a factor of 50% after culturing for 6 h, and then the rate of cell proliferation was almost constant for 60 h. The cell proliferation studies show that the cytotoxicity of PbS QDs is not significant at concentrations less than 5 nM. In the fluorescence imaging of cerebral blood vessels in mice, high concentrations (3 μM) of PbS QDs (100–200 μL) was used for intravascular injection of the QDs. However, acute toxicity of the PbS QDs was not observed, where the acute toxicity may have induced the deaths of mice.

### 2.3. Optical Properties of Mouse Brain in the Visible and NIR Region

**Figure 3:**
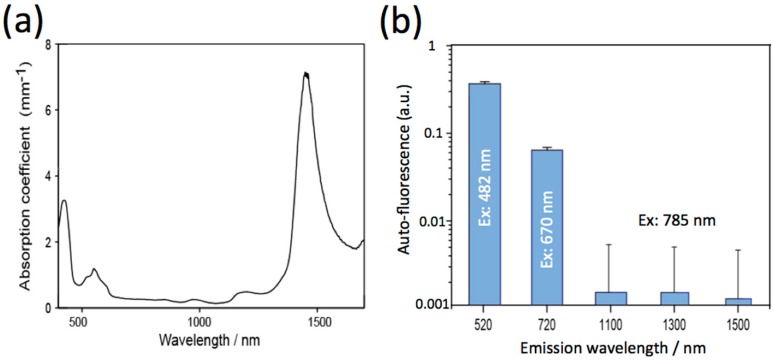
(**a**) Absorption spectrum of a mouse brain; (**b**) Autofluorescence of a mouse brain. The fluorescence at 520 and 720 nm was obtained by excitation at 482 and 670 nm, respectively. The fluorescence at 1110, 1300, and 1500 nm was obtained by excitation at 785 nm.

To get clear NIR fluorescence images of mouse brains, excitation wavelengths should be optimized to reduce the absorption and autofluorescence by the brain tissue. The absorption spectrum of a mouse brain showed that the absorption of light by the brain tissue at the NIR wavelength region (700 to 1400 nm) is very low (Figure 3a). This region is called the optical window in living tissues. The region from 700 to 1000 nm is called the first optical window, while the region from 1000 to 1400 nm is called the second optical window [16]. Absorption at the visible region less than 600 nm is attributed to intrinsic chromophores such as flavin and hemoglobin. The intense absorption at around 1500 nm is due to absorption by water in the brain tissue. Autofluorescence of the mouse brain strongly depends on the wavelength of excitation (Figure 3b). It should be noted that the intensities of autofluorescence at 1100, 1300, and 1500 nm excited at NIR light (785 nm) is at least 50 times lower than that (520 and 670 nm emission) excited by visible light at 482 nm and 670 nm. Based on these findings, we used a 1100 nm-emitting PbS QDs (QD1100) as a NIR fluorescence probe (excitation: 785 nm) for in vivo fluorescence imaging of cerebral blood vessels.

### 2.4. NIR Fluorescence Imaging of Cerebral Blood Vessels in Mice

**Figure 4:**
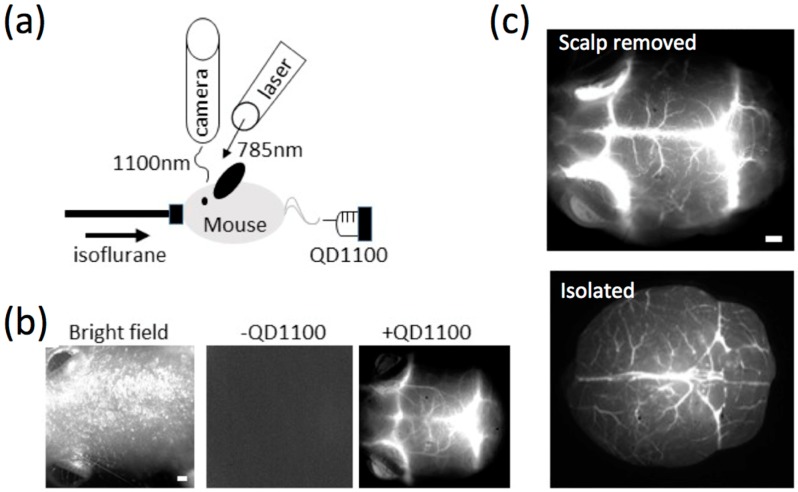
(**a**) Experimental setup for NIR fluorescence imaging of cerebral blood vessels. An anesthetized mouse was administered QD1100 in a caudal vein. An optical laser (785 nm wavelength) was used as an excitation light source, and NIR fluorescence was detected with an InGaAs camera; (**b**) Imaging of a mouse head. Bright field image (**left**), NIR fluorescence image without QD administration (**middle**), and the NIR fluorescence image with QD administration (**right**). Scale bar: 1 mm; (**c**) NIR fluorescence images of cerebral blood vessels. Upper: fluorescence image after scalp removed, lower: fluorescence image after isolation. Scale bars: 1 mm.

**Figure 5:**
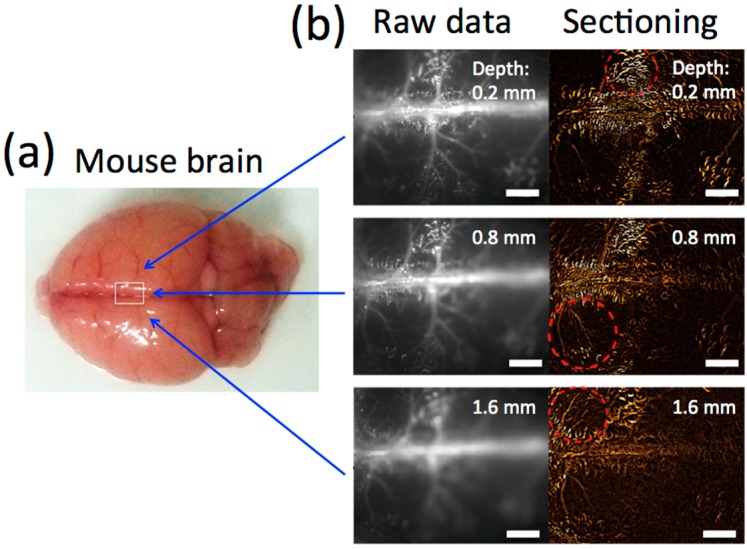
(**a**) Bright field image of a mouse brain isolated; (**b**) Raw fluorescence images and sectioning images at depths of 0.2 mm, 0.8 mm, and 1.6 mm from the surface. The sectioning image was obtained from a raw image minus its previous and next image. Red circles with dotted lines show cerebral blood vessels appearance after sectioning. Scale bars: 1 mm.

NIR fluorescence (>1000 nm) of QD1100 from a mouse head was detected after intravascular injection of QD1100 from a caudal vein (Figure 4a). Before administration of QD1100, only very weak NIR fluorescence signals (>1000 nm) were detected (Figure 4b, middle), because of the very low background autofluorescence (see, Figure 3b) in this NIR region. When QD1100 was intravascularly injected into the mouse, NIR fluorescence signals (>1000 nm) from the mouse head immediately increased, and the vascular structure of cerebral blood vessels was clearly visualized (Figure 4b, right). To confirm that the image shows cerebral blood vessels through mouse scalp and skull, we observed NIR fluorescence images of the mouse brain after removal of its scalp skin (Figure 4c, upper image). In addition, we observed NIR fluorescence images of an isolated mouse brain (Figure 4c, lower image). These images show similar vascular structures with that (Figure 4b, right) which is non-invasively imaged, suggesting that the intravascular injection of QD1100 in a caudal vein can non-invasively visualize cerebral blood vessels through mouse scalp and skull. Visualization depth for the cerebral blood vessels was checked by measuring z-stacked images for the isolated brain (Figure 5). In our NIR imaging system, maximum depth for the visualization of the fine structure of cerebral blood vessels was determined to be ca. 1.6 mm.

### 2.5. NIR Fluorescence Imaging of Lipopolysaccharide-Induced Thrombosis in Cerebral Blood Vessels in Living Mice

**Figure 6:**
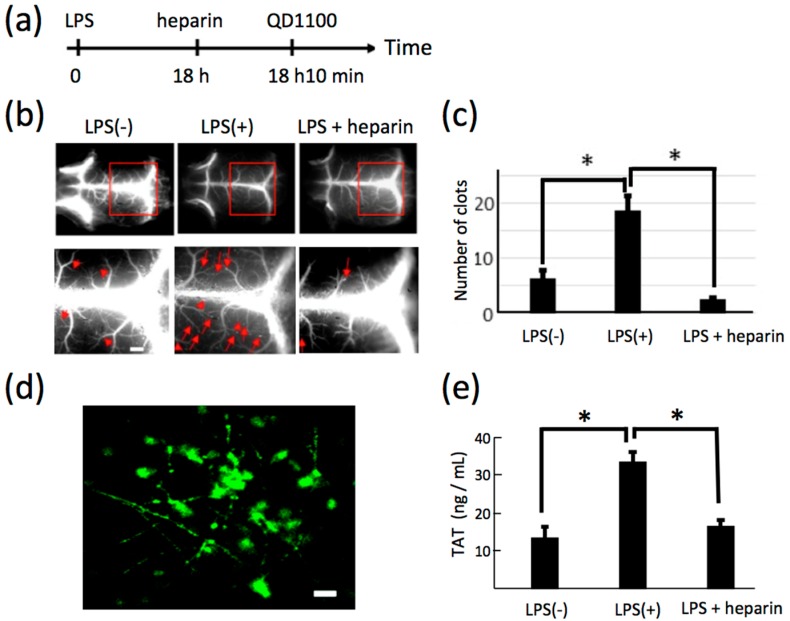
(**a**) Time course of experimental procedure for lipopolysaccharide (LPS) and heparin administration; (**b**) NIR fluorescence images (>1000 nm) of cerebral blood vessels before and after administration of LPS (LPS(−) and LPS(+)), and the image following additional administration of heparin (LPS + heparin), with scalp removed. Lower panel shows the magnification of the images shown by red rectangles. Red arrowheads show clots. Scale bars: 1 mm; (**c**) Statistical analyses of the clots in the cerebral vessels. *: *p* < 0.05, number of mice: LPS(−): *n* = 5, LPS (+): *n* = 5, LPS + heparin: *n* = 3; (**d**) Immunofluorescence staining of LPS-treated cerebral blood vessels, where anti-fibrinogen antibody (Alexa Fluor 488) was used for staining of fibrinogen. Fibrinogen helps the formation of blood clots. Scale bar: 10 μm; (**e**) ELISA assays for thrombin–antithrombin complex (TAT) in blood plasma. *: *p* < 0.05, number of mice: LPS(−): *n* = 5, LPS(+): *n* = 5, LPS + heparin: *n* = 3.

We examined whether a pathophysiological state of blood vessels in sepsis can be visualized by NIR fluorescence imaging. To induce thrombosis in cerebral blood vessels, lipopolysaccharide (LPS) was intraperitoneally injected to mice. After 18 h, heparin (an inhibitor of blood coagulation [34,35,36]) was intravenously injected to the mouse. Then, QD1100 was administered 10 minutes before NIR fluorescence imaging (Figure 6a). NIR fluorescence imaging of the mouse head showed LPS-induced thrombosis in cerebral blood vessels (Figure 6b). The magnified images with the scalp removed (Figure 6b, lower panel) of blood vessels show that septic clots (i.e., thrombosis) were increased by the administration of LPS, and heparin resulted in a suppression of the number of clots (Figure 6c). Figure 6d is the immunohistochemistry for an LPS-administered brain slice, showing the formation of clots in cerebral blood vessels.

Next, blood coagulation was quantified by enzyme-linked immunosorbent assays (ELISA) (Figure 6e). Thrombin–antithrombin complex (TAT) is a valid biomarker for disseminated intravascular coagulation [37]. After administration of LPS, averaged TAT values were significantly increased, and the level of TAT was recovered by heparin administration. This finding suggests that disseminated intravascular coagulation was induced in an LPS-induced mouse model of sepsis.

### 2.6. Significant Change in the Number of LPS-Induced Thrombi in Isolated Brains

**Figure 7:**
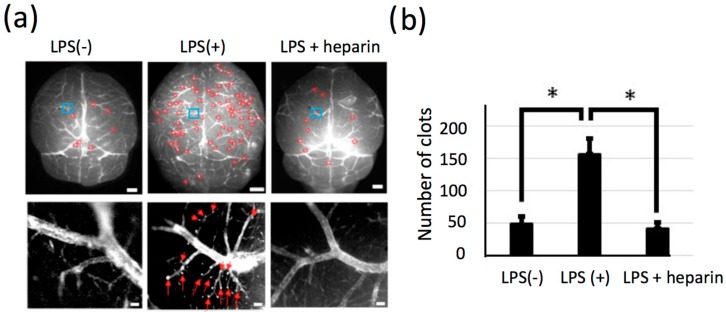
(**a**) Upper panel: NIR fluorescence images (>1000 nm) of cerebral blood vessels of isolated mouse brains. Left: LPS(−), Middle: LPS(+), Right: LPS + heparin. Red circles: clots. Blue squares: region of interests for the magnified views of lower panels. Scale bars: 1 mm. Lower panel: magnified NIR fluorescence image of cerebral blood vessels at the bregma. Red arrows: clots. Scale bars: 100 μm; (**b**) Number of clots for each mouse. *: *p* < 0.05, number of mice: LPS(−): *n* = 5, LPS(+): *n* = 5, LPS + heparin: *n* = 4.

For the statistical analysis of LPS-induced thrombosis in cerebral blood vessels, NIR fluorescence imaging for isolated brains was conducted. A significant increase in the number of clots was clearly found after the administration of LPS (Figure 7). High magnification images (Figure 7a, lower panel) showed blood coagulation in the capillaries of cerebral blood vessels. The number of thrombi in the capillaries was decreased by 60% after the administration of heparin (Figure 7b). By using the NIR fluorescence of QD1100, the pathological state of the mouse brain induced by LPS was clearly visualized. In the preclinical study, it was reported that QDs were useful to label and were applied to flow cytometry analyses of thrombosis in the blood plasma of human subjects [38]. In addition, Averett reported that QDs can label a clot in the fibrin network only in the presence of matrix metalloprotease and thrombin [39]. These reports support our findings that QDs are useful to label clots in blood vessels of thrombotic condition.

## 3. Experimental Section

### 3.1. Chemicals

Lead(II) chloride, oleylamine, and oleic acid were purchased from Wako Pure Chemical Industries (Tokyo, Japan). Hexamethyldisilathiane and tributylphosphine (TBP) were purchased from TCI Chemicals (Tokyo, Japan). Mercaptoundecanoic acid (MUA) and potassium tert-butoxide were purchased from Sigma-Aldrich ((St. Louis, MO, USA). Other chemicals used were of analytical grade.

### 3.2. Synthesis and Characterization of NIR Fluorescent PbS QDs (QD1100)

PbS QDs (QD1100) were synthesized by the method described elsewhere [6]. A typical synthetic procedure was as follows: lead(II) chloride (278 mg) was dissolved in a mixture of 5 mL oleylamine and 1 mL oleic acid in a three-necked flask at room temperature. The solution was heated to 100 °C under an argon atmosphere. To this solution, 0.5 mL of a sulfur precursor solution (0.5 mL of hexamethyldisilathiane and 9.5 mL of TBP) was added dropwise under vigorous stirring. The formation of PbS QDs was monitored by observing their emission spectra, which shifted to longer wavelengths by increasing the amount of the sulfur precursor solution. When 1100 nm emission of PbS QDs was obtained, the solution was quickly cooled to 60 °C. Then, methanol was added to the solution to precipitate the QDs. To 1 mL of the tetrahydrofuran solution of PbS QDs (1 μM), 1 mL of MUA solution (50 mg/mL in tetrahydrofuran) was added under stirring. To this solution, 0.1 mL of aqueous solution (50 mg/mL of potassium *t*-butoxide) was added to precipitate the MUA-coated PbS QDs. The PbS QD precipitates were separated by centrifugation and dissolved to water. An aqueous solution of PbS QDs was passed through a 0.22 μm membrane filter and centrifuged at 15,000 *g* for 5 min to remove aggregated PbS QDs.

Transmission electron microscopy (TEM) of QD1100 was conducted by H-800 (Hitachi, Tokyo, Japan). The fluorescence spectra were measured by a NanoLog spectrofluorometer (Horiba, Kyoto, Japan). Hydrodynamic size of QD1100 was determined by measuring dynamic light scattering (Marvern Nano-ZS).

### 3.3. Biocompatibility of QD1100

To evaluate the cytotoxicity of QD1100, the effects of the QDs on a cell proliferation test was examined by using the Celigo Imaging Cell Cytometer (Nexcelom Bioscience LLC, Lawrence, MA, USA). HeLa cells were plated at 6000 cells/well in 96-well plates. After culturing HeLa cells overnight, QD1100 was added to every well at a final concentration of 0–50 nM. The number of cells in each well was counted with Celigo after 0, 7, 24, 48, and 60 h.

### 3.4. Mice

Hairless four week-old Hos:HR-1 young mice were purchased from Japan SLC Inc. (Shizuoka, Japan). To reduce the autofluorescence, special feed without alfalfa (Oriental yeast, Tokyo, Japan) was fed to all mice until the onset of the experiment. Experiments were conducted after one week of feeding. All of the following experimental procedures on mice were conducted under anesthesia using a small animal anesthetizer (MK-A110D, Muromachi kikai, Tokyo, Japan) with isoflurane. A mouse model of sepsis was conducted as described elsewhere [40]. To induce sepsis, mice were intraperitoneally treated with lipopolysaccharide (LPS, 1.0 mg per mouse) from *Escherichia coli* (055:B5, Sigma-Aldrich). QD1100 (3 μM) and heparin (100 units, Mochida Pharm., Tokyo, Japan) per mouse were injected in a caudal vein. After the recording, mice were sacrificed under deep anesthesia, and brain tissues were dissected. All of the experimental procedures were done in accordance with the declaration of Helsinki. Prior to the onset of experiments, the animal experimental plan was approved by the ethics committee of Riken (approved No. QAH27-03).

### 3.5. Optical Properties of Brain Tissue

Brain tissue extracted from the Hos: HR-1 mice was separately enclosed by two glass cover slips. NIR fluorescence images were taken with HCimage system (Hamamatsu photonics, Hamamatsu, Japan) built in MVX microscopy. For quantitative comparisons of autofluorescence at different emission wavelengths, image intensities were corrected for photon counts per unit excitation power and unit bandwidth of the emission filter. For diffuse transmittance and collimated transmittance measurements, the samples were located at the entrance of the integrating sphere where the round cover across the optical path of the collimated transmittance was removed. For diffuse reflectance measurements, the sample was located at the exit of the integrating sphere. Diffuse and collimated transmittance and diffuse reflectance spectrums of tissue samples were measured using a JASCO V-670 spectrophotometer with an integrating sphere (JASCO). The attenuation coefficient was calculated from the collimated transmittance. The absorption coefficient was calculated from the diffuse transmittance and reflectance.

### 3.6. Microscopy System for Cerebral Vessel Imaging

Brain vascular recording was performed with the HCimage system as described elsewhere [6]. In brief, mice were fixed with a head holder for mice (SGM-4, Narishige, Tokyo, Japan) on a stage set of the MVX microscope (Olympus, Tokyo, Japan). All mice were anesthetized with isoflurane containing air mixture during the recording. Body temperatures of mice were maintained at 37 °C with a heat pad. The heads of mice were illuminated using a 785 nm solid laser (BW TEK, San Francisco, CA, USA) with a laser power (40 mW/cm^2^). The fluorescence emission was passed through a long-pass filter (>1000 nm) and recorded with an InGaAs camera (C10633-34, Hamamatsu photonics, Hamamatsu, Japan). The same region of interest (R.O.I) among mice groups was recorded under the same experimental conditions (exposure time: 5 s for laser, objective lens: ×0.63, 2.0, 6.3 for microscope). For clot analysis, the threshold of recorded data was calculated and the number of clots appearing on each picture was calculated with Image J software (ver. 1.49t, NIH, Bethesda, MD, USA).

### 3.7. Immunofluorescence Staining of Brain Tissues

A slice of brain tissue (thickness, 1 mm) was prepared from a septic mouse, and the slice was stained with Alexa Fluor 488 conjugated anti-fibrinogen alpha chain antibody (Bioss, Woburn, MA, USA). The experimental procedure for tissue imaging is described elsewhere [40]. Fluorescence images of the brain tissue slice were taken with a fluorescence microscope (Keyence, Osaka, Japan).

### 3.8. Enzyme-Linked Immunosorbent Assays (ELISA)

Blood coagulation factors in the blood plasma across mice groups (LPS(−), LPS(+), and LPS + heparin) were examined by the ELISA Kit for mouse thrombin–antithrombin complex (CUSABIO, College Park, MD, USA). Experiments were conducted according to the manufacturer’s protocol. Blood plasma (700 + 100 μL) were obtained from the inferior vena cava of mice, and stored at −80 °C before use.

### 3.9. Statistical Analyses

The recorded data was analyzed with Fiji Image image processing package with open source architecture built on Image J. The analyzed data were shown as means ± SEM. The significance of differences among mice groups was assessed with one-way ANOVA. Values of *p* < 0.05 determined statistical significance for all analyses.

## 4. Conclusions

In this study, we demonstrate that QD1100 can be used as a NIR fluorescent probe for the non-invasive visualization of cerebral blood vessels in living mice. In addition, QD1100 can display the microstructure of thrombi in septic mice induced by LPS. Although single-walled carbon nanotubes can act as a NIR fluorescent probe for visualization of blood flow in the mouse brain [14], a valid NIR probe for the evaluation of the disease state of the brain has been required. Owing to the high brightness of QD1100, we could non-invasively observe the pathological state of the brain (cerebral venous thrombosis) in septic mice. Septic encephalopathy results in blood coagulation in cerebral blood vessels. The observation of blood coagulation (thrombosis) in initial melanoma metastasis in mouse lungs has been reported using bioimaging with QDs [41]. The pathophysiology of septic encephalopathy is gradually becoming possible to explain by molecular mechanisms [24,42,43]; however, the diagnosis of septic encephalopathy remains difficult to determine. NIR fluorescence imaging with highly-fluorescent PbS QDs would be useful for the study of the pathophysiology of cerebral blood vessels which induce brain dysfunction.
